# Intrinsic Learning Rather than External Difficulty Dominates Decision Performance: Integrated Evidence from the Drift-Diffusion Model and Random Forest Analysis

**DOI:** 10.3390/bs16020300

**Published:** 2026-02-20

**Authors:** Yanzhe Liu, Qihan Zhang

**Affiliations:** 1 Key Research Base of Humanities and Social Sciences of the Ministry of Education, Academy of Psychology and Behavior, Tianjin Normal University, Tianjin 300387, China; 2 Faculty of Psychology, Tianjin Normal University, Tianjin 300387, China

**Keywords:** learning, decision-making, random forest, drift-diffusion model

## Abstract

Previous studies have emphasized the role of task difficulty in decision performance while relatively neglecting the decision maker’s subjective initiative and intrinsic learning process during task execution. This study manipulated the rule hierarchy factor, which reflects external task difficulty, and the block factor, which reflects the accumulation of intrinsic learning, and used analysis of variance (ANOVA), the drift-diffusion model (DDM), and random forest algorithms to systematically examine how task difficulty and learning jointly influence decision behavior and its underlying mechanisms. A total of 40 participants were recruited, and after strict exclusion criteria were applied, 34 valid datasets were included in the final analysis. The results showed that although rule hierarchy had a significant impact on decision performance in the early stage of the task (the first two blocks), this effect gradually diminished as task repetitions increased. Furthermore, the results revealed a clear dissociation in predictive mechanisms: intrinsic cognitive factors (specifically, evidence accumulation efficiency and decision bias) were the primary predictors of decision accuracy, whereas external task difficulty (rule hierarchy) acted as the dominant predictor for decision speed (reaction time). These findings provide a new perspective for understanding the dynamic relationship between external task demands and intrinsic learning processes, highlighting the necessity of distinguishing between accuracy and speed metrics in personalized education, training, and human–computer interaction design.

## 1. Introduction

In the field of decision-making research, task difficulty is generally regarded as one of the key factors influencing decision performance (Jacobs & Gaver, 1998; Newell & Shanks, 2014). This relationship can be systematically explained by Cognitive Load Theory, which posits that individuals have limited working memory capacity. When task complexity increases substantially and the total amount of information to be processed exceeds the capacity limit of working memory, cognitive processing efficiency declines, leading to longer decision reaction times and higher error rates (Sweller, 2023, 2011). Rule hierarchy serves as an effective indicator of task difficulty (Harada et al., 2022; Van Maanen et al., 2021). It refers to the hierarchical structure defined by the level of abstraction, logical complexity, and cognitive integration required by the task rules: lower hierarchies correspond to simple stimulus–response associations, whereas higher hierarchies involve multi-rule conditional judgments (Zhu & Han, 2022). As the rule hierarchy increases, the demand for information integration grows, resulting in higher cognitive load, prolonged reaction times, and reduced accuracy. Previous research has consistently confirmed this phenomenon (Harada et al., 2022; Nee & Brown, 2012; Koechlin & Summerfield, 2007). Therefore, rule hierarchy, as an external task attribute, directly affects individuals’ decision performance.

However, this research perspective, which centers on “external tasks,” has theoretical limitations. Its key shortcoming lies in neglecting the decision maker’s subjective initiative and intrinsic learning process during task execution. According to Learning Curve Theory (Harris, 2022; Mazur & Hastie, 1978), as task repetitions accumulate, individuals’ performance systematically improves. This improvement is not a passive adaptation to external tasks but an active process through which the cognitive system continuously optimizes information processing strategies and enhances resource allocation efficiency (Markant et al., 2016). The block factor in experiments is typically used to examine how cumulative execution and learning influence individual performance (van der Kleij et al., 2019). Studies have found that as the number of blocks increases, performance improves accordingly, manifesting as shorter reaction times and higher accuracy (Moisello et al., 2009; Han et al., 2024).

It is thus evident that an individual’s decision performance should be jointly determined by both external task factors and intrinsic cognitive processes (Van Maanen et al., 2021; Li et al., 2024). However, previous studies have typically examined their effects from a single perspective. For example, Verbruggen et al. (2018) focused on how rule hierarchy affects behavioral performance but neglected the cumulative learning effects that accompany task experience, such as increasing blocks. Other studies have examined changes in reaction time and accuracy as a function of blocks or practice frequency but failed to differentiate the cognitive processing demands required by rule complexity or rule hierarchy (Suzuki et al., 2022). Moreover, even studies that considered both rule hierarchy and block factors often limited their analyses to the behavioral level (reaction time and accuracy), lacking deeper exploration of the underlying cognitive processes and thus providing no direct evidence for the active construction of cognition. For instance, although Karayanidis et al. (2023) manipulated high- and low-hierarchy rule switching in a task-switching paradigm, they only reported behavioral effects without examining the associated internal cognitive processing. Prior research also did not clarify which factor—rule hierarchy or cumulative practice across blocks—plays a more dominant role in shaping decision performance. Therefore, the effects and underlying mechanisms of rule hierarchy and block on decision performance require further investigation.

To address these issues, the present study designed a targeted experimental task and adopted a progressive data analysis approach. At the experimental design level, rule hierarchy (with multiple rule conditions) was manipulated to systematically characterize varying levels of external task difficulty, providing a clear variable basis for analyzing its impact on decision performance. A multi-block task structure was employed to dynamically track changes in both behavioral performance and internal processes as task repetitions increased under different rule hierarchy conditions, thereby capturing how intrinsic learning progression modulates rule processing over time. At the data analysis level, this study integrated three complementary analytical tools—analysis of variance (ANOVA), the drift-diffusion model (DDM), and random forest algorithms—to construct a multidimensional analytical framework. ANOVA was used to examine the effects of rule hierarchy and block on behavioral outcomes. The DDM estimated key parameters—drift rate (v, reflecting evidence accumulation speed and cognitive processing efficiency), boundary separation (a, reflecting decision caution), and non-decision time (t_0_, reflecting perceptual encoding and motor preparation time)—to reveal the internal cognitive mechanisms underlying the effects of rule hierarchy and block. The random forest approach further synthesized the experimental design and modeling results to explore the relative importance of indicators within “external task” and “intrinsic cognition” in predicting decision performance.

Cognitive Load Theory has long emphasized how external task complexity affects performance through the limitations of working memory capacity, manifesting as longer reaction times and reduced accuracy (Paas & Van Merrienboer, 2020; Sweller, 2011, 2023). However, this theory overlooks the role of active learning within the individual during task execution. Learning Curve Theory, in contrast, proposes that with accumulated experience, the cognitive system actively optimizes processing strategies and improves resource allocation efficiency, thereby systematically enhancing task performance (Harris, 2022; Mazur & Hastie, 1978). Previous studies have shown that as the number of blocks increases, reaction times shorten and accuracy improves, reflecting a clear learning effect (Moisello et al., 2009; Han et al., 2024). Based on these findings, the present study proposes a central hypothesis: decision performance depends more on intrinsic cognitive processes than on external task difficulty alone. Specifically, although rule hierarchy (task complexity) may initially produce large differences in reaction time and accuracy, these differences are expected to gradually diminish with increasing experience, reflecting the mitigating effect of learning on external difficulty. This study thus provides a solid foundation for deepening the understanding of the relationship among task difficulty, learning processes, cognitive mechanisms, and decision performance, while also offering practical insights for the personalized optimization of educational training and human–computer interaction.

## 2. Methods

### 2.1. Participants

Participants were recruited from Tianjin Normal University, yielding a total of 40 participants (19 males and 21 females). The average age was 19.71 years (range: 18–22 years). All participants were native Chinese speakers. Their uncorrected or corrected vision was normal, and none exhibited color blindness or color weakness. In addition, all participants were able to clearly distinguish the textures, borders, colors, and patterns used in this study and reported no discomfort when viewing the experimental materials. All participants provided written informed consent prior to the formal experiment and received monetary compensation after completing the study. This experiment was approved by the Ethics Committee of Tianjin Normal University (No. 2023112302).

### 2.2. Experimental Design

The experiment adopted a 3 (Rule Hierarchy: Hierarchy 1, Hierarchy 2, Hierarchy 3) × 5 (Block: 1, 2, 3, 4, 5) within-subjects factorial design. Rule Hierarchy served as the external task difficulty manipulation, while Block served as a repeated-measures temporal factor to assess intrinsic learning effects. The dependent variables included participants’ keypress reaction time and response accuracy.

### 2.3. Experimental Task

The experiment consisted of two stages: a learning stage and an experimental stage. Participants were required to practice the experimental task and achieve an accuracy rate above 80% during practice in order to proceed; those who did not meet the criterion were asked to repeat the practice session. Only after successfully completing the learning stage could participants proceed to the experimental stage.

#### 2.3.1. Learning Stage

**Figure 1:**
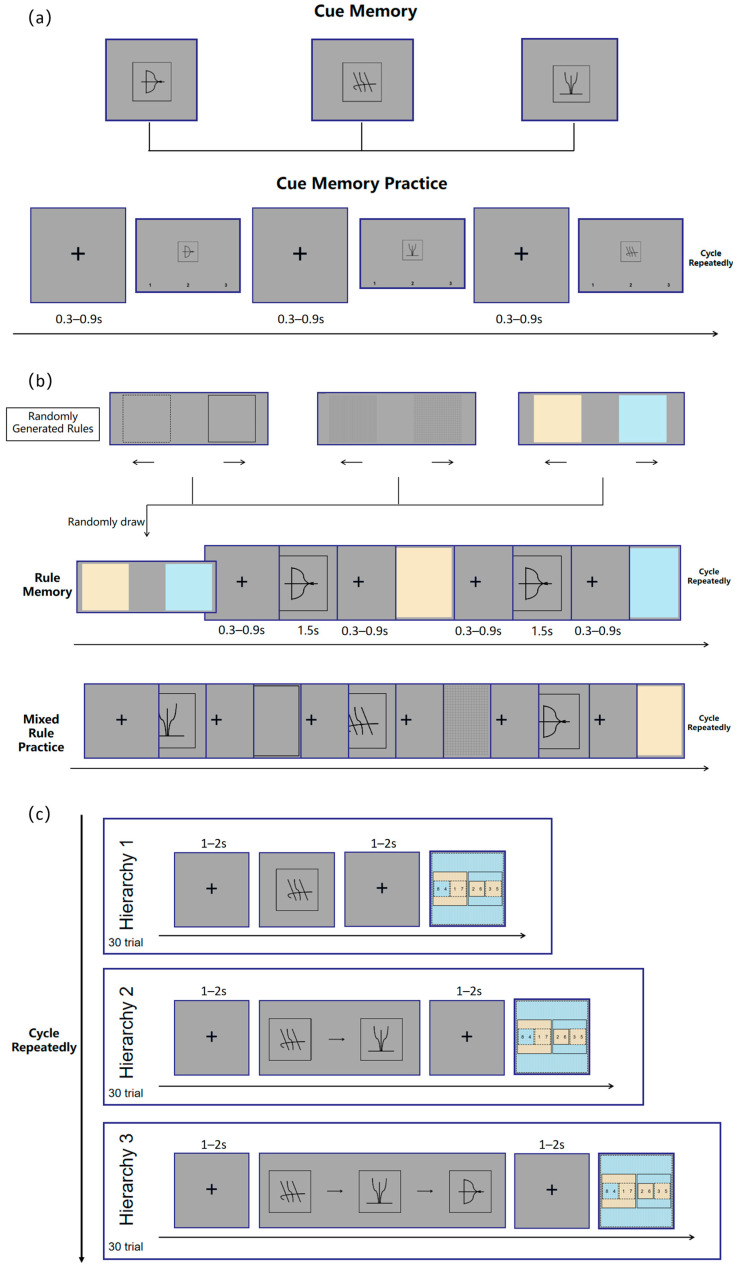
Experimental flowchart. (**a**) The Cue Memory and Cue Memory Practice phases, where participants learned to associate abstract symbols with specific judgment rules; (**b**) The Rule Memory and Mixed Rule Practice phases, illustrating the specific feature dimensions (color, texture, border) and the randomized trial sequence used to reinforce rule acquisition; (**c**) The formal Experimental Stage, depicting the flow of a single trial under Hierarchy 1 (single cue), Hierarchy 2 (two sequential cues), and Hierarchy 3 (three sequential cues) conditions, where participants analyzed a composite target figure composed of seven nested squares based on the preceding rule cues.

**Figure 2:**
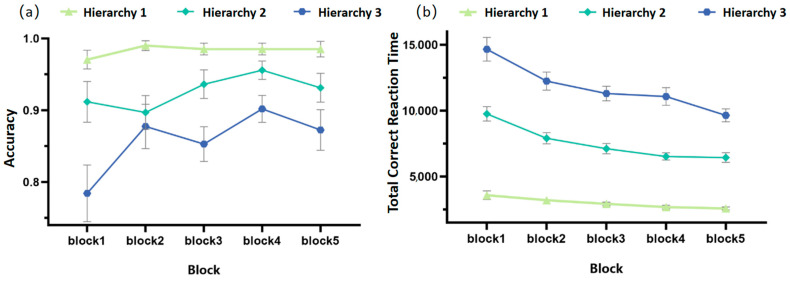
Changes in Accuracy Rate and Total Correct Reaction Time Over Time. (**a**) The trajectory of response accuracy under three Rule Hierarchy conditions (Hierarchy 1, 2, and 3) across five blocks; (**b**) The trajectory of total correct reaction time under three Rule Hierarchy conditions across five blocks.

(1) Cue Memory. Participants first learned three cue symbols and their corresponding judgment rules, with the symbol–rule pairings assigned randomly. They then entered the practice phase for cue–rule associations. During this phase, the cue symbols learned previously were presented one by one at the center of the screen. Keys 1, 2, and 3 represented the three rules, respectively. Participants were required to quickly and accurately identify the meaning associated with each cue symbol and make the correct keypress response. If a participant responded incorrectly, the corresponding cue would reappear on the screen along with the instructional message from the cue memory phase, prompting the participant to memorize it again. A fixation cross was presented for 0.3–0.9 s between trials, as illustrated in Figure 1a.
(2) Rule Memory. A cue image was first presented on the screen, and the accompanying instructions informed participants of the rule corresponding to that cue. Participants were required to memorize the attentional directions associated with the two features under that rule. They then entered the practice phase for the corresponding rule. In each trial, the cue symbol for the rule appeared first on the screen, prompting the participant to recall the rule it represented. Next, one of the features under that rule was randomly presented, and participants were asked to judge its attentional direction (left or right) according to the rule and press the corresponding key (left or right arrow key). The stimulus disappeared immediately after the response. If the response was incorrect, the system provided corrective feedback. Specifically, for the color rule, the two features were blue and orange; for the texture rule, the features were vertical lines and crosshatch textures; and for the border rule, the features were solid and dashed borders. The attentional direction assigned to each feature was randomized, with one feature corresponding to the left direction and the other to the right. For example, when the selected rule was the color rule and its cue image corresponded to the “bow and arrow” symbol (as shown in the top-left corner of the third image in the first row of Figure 1a), orange might correspond to the left and blue to the right. A fixation cross lasting 0.3–0.9 s was presented between trials, as illustrated in Figure 2. This procedure was repeated three times (once for each rule), with 20 trials per rule (10 trials per feature). Each session involved one rule and one cue image. The order of rules was randomized, and all rules and features were sampled without replacement to ensure the independence and randomness of each trial.
(3) Mixed Rule Practice. This phase tested participants’ memory of all three rules. Each feature was presented in five trials, resulting in 10 trials per rule. The three rules were randomly intermixed, producing a total of 30 trials. The trial procedure was identical to that used in the rule memory phase, and the overall process is shown in Figure 1b.
(4) Experimental Practice. Participants were first informed of the content and procedures of the formal experiment through on-screen instructions. They then proceeded to a practice session, which followed the same procedure as the formal experiment (see the following section for details), except that the number of trials was smaller and feedback was provided, allowing participants to know whether their responses were correct.

#### 2.3.2. Experimental Stage

In the formal experiment, after a fixation cross lasting 1–2 s, the screen first displayed one to three cue images (the cue screen). Participants were required to recall the corresponding rules indicated by the cue images and determine the hierarchical relationship among the rules according to the arrows shown on the cue screen. They pressed a designated key to indicate that they had completed the rule processing. After another 1–2 s of fixation, a composite figure screen appeared, showing a figure composed of seven nested squares, each containing three attributes: color, texture, and border. Participants were instructed to analyze the composite figure according to the rules indicated by the cue images. The analysis sequence proceeded from the outermost large square to the middle and then to the innermost small square.

When only one cue image appeared, it represented a single-hierarchy rule condition. Participants needed to apply that rule to the outermost large square, identify its corresponding feature, and press the associated numeric key. When two cue images appeared, representing a two-hierarchy rule condition, participants first applied the rule indicated by the first cue to the outermost square and pressed the first numeric key, then used the identified feature to determine which middle square to observe next, applied the second cue’s rule to that square, and pressed the second numeric key. When three cue images appeared, representing a three-hierarchy rule condition, participants sequentially applied all three rules: they first used the rule from the first cue to analyze the outermost square and pressed the first numeric key, then determined which middle square to inspect and pressed the second numeric key, then applied the second cue’s rule to the middle square to determine which small square to observe, and finally applied the third cue’s rule to the innermost square to determine the final numeric response.

To investigate the dynamic changes in decision performance, the formal experiment was divided into 5 sequential Blocks. The total of 90 trials (30 trials per Rule Hierarchy) was equally distributed across these 5 Blocks. Consequently, each Block consisted of 18 trials (6 trials for each of the three Rule Hierarchies). To control for order effects while tracking learning, the presentation order of the Rule Hierarchy conditions was randomized within each Block. Participants were given adequate rest between blocks. The detailed experimental procedure is illustrated in Figure 1c.

### 2.4. Experimental Questionnaires

Color blindness and color weakness test. Before the experiment, participants were screened for color blindness and color weakness using Atlas of Color Blindness Inspection (Yu et al., 1996). During the screening, 2 images were selected from the atlas, and participants were required to correctly answer the content contained in the images. If a participant could answer all questions correctly, they were considered to have passed the color blindness and color weakness test.

Self-designed experimental material discrimination questionnaire. This questionnaire consists of 6 questions, covering question types related to cue diagrams, colors, textures, and borders. For each question, 2 images of the same type (both are materials used in the experiment) were displayed on the screen, and participants needed to press a key to judge the content corresponding to the images. If each image could be clearly distinguished, it indicated that the participant could effectively discriminate the key materials in the experiment. This process aimed to ensure that participants had the ability to accurately identify the experimental materials, so that the subsequent experiment could be carried out smoothly.

### 2.5. Experimental Procedure

The questionnaires for this experiment were self-designed using the TClab online experimental platform (https://www.testcloudlab.com/ (accessed on 19 January 2026)). After being released online, participants were required to complete the questionnaires using a computer or mobile phone. The experimental program was written using MATLAB software (version R2022b) and the PsychToolbox-3 toolbox. The specific procedure is as follows: (1) Participants filled out the informed consent form, and completed the demographic information survey and experimental questionnaires. The questionnaire content included basic personal information such as age and gender, as well as the Color Blindness and Color Weakness Test, and the Self-Designed Experimental Material Discrimination Questionnaire. Only when participants’ results met all the experimental requirements could they proceed to the experimental session. (2) Completion of the experimental task. Participants could only enter the formal experiment after passing the learning phase.

### 2.6. Data Analysis

#### 2.6.1. Data Preprocessing

Before data analysis, outliers with excessively short or long reaction times were eliminated, and data from participants who failed to make hierarchical responses as required or whose accuracy rate in the learning phase did not reach 80% were removed.

#### 2.6.2. Analysis of Variance (ANOVA)

To examine the effects of Rule Hierarchy and block on accuracy rate and reaction time, a 3 (Rule Hierarchy: Hierarchy 1, Hierarchy 2, Hierarchy 3) × 5 (block: 1, 2, 3, 4, 5) two-factor repeated-measures Analysis of Variance (ANOVA) was conducted.

#### 2.6.3. Drift-Diffusion Model (DDM)

(1) Model Construction

To examine the effects of Rule Hierarchy and Block on the decision process, four types of competing models were constructed based on the Drift-Diffusion Model (DDM). The DDM decomposes performance into four latent parameters: drift rate (v), representing the efficiency of evidence accumulation; boundary separation (a), reflecting the threshold for decision caution; non-decision time (t_0_), accounting for sensory encoding and motor response; and starting point proportion (z), which signifies the relative bias toward a decision boundary. Specifically, the starting point proportion (z) is determined by the ratio of the absolute starting point bias (Z_0_) to the boundary height (a). In this study, the starting point bias (Z_0_)—and consequently the proportion (z)—was allowed to vary across conditions. This specification was based on the experimental design where the Rule Cue was presented prior to the target stimulus (with a 1–2 s interval), providing a preparation window that allowed participants to proactively adjust their decision bias according to the anticipated Rule Hierarchy before evidence accumulation began. The models were constructed as follows:

Model 1: DDM with Main Effect of Rule Hierarchy, which only considers the effect of Rule Hierarchy on DDM parameters.

Model 2: DDM with Main Effect of Block, which only considers the effect of Block on DDM parameters.

Model 3: DDM with Dual Main Effects, which considers the main effects of both Rule Hierarchy and Block simultaneously.

Model 4: DDM with Dual Main Effects and Interaction, which further incorporates the interaction effect between Rule Hierarchy and Block on the basis of Model 3.

(2) Parameter Estimation

A Hierarchical Bayesian parameter estimation method written in custom MATLAB code was used for parameter estimation, adopting the adaptive random walk Metropolis–Hastings sampling strategy (MCMC). This hierarchical framework allowed for the simultaneous estimation of group-level hyperparameters and subject-level parameters, ensuring robust estimation stability even with limited trial numbers per condition. Uninformative uniform priors were used for all parameters to minimize bias and allow the data to drive the posterior distributions. To improve the reliability of estimation, 3 independent sampling chains were set. For the modeling of Rule Hierarchy and Block, the MCMC chains had a total of 3000 iterations, with the first 1500 iterations serving as the burn-in period to eliminate the influence of initial values. To balance sampling efficiency and convergence, the local acceptance rate was calculated every 200 iterations, and the step size was adaptively adjusted accordingly to make the acceptance rate approach the target value of 0.30. The final acceptance rate stabilized in the range of 0.25–0.35, meeting the statistical reliability criteria for model fitting. To ensure numerical stability and parameter interpretability, constrained transformation was applied to the parameter space: boundary height a > 0.1, non-decision time t_0_ ∈ (0, minimum reaction time), and starting point proportion z ∈ (0, 1).

After model fitting, the Gelman–Rubin diagnostic index (R-hat) was used to evaluate the convergence of MCMC. The maximum R-hat value under all conditions was <1.05, indicating that all chains converged well. To further validate model adequacy, we performed a Posterior Predictive Check (PPC) by simulating decision data using the estimated posterior means. The simulated reaction time distributions matched the observed empirical data well, supporting the interpretability of the parameters. Subsequently, the posterior distributions of each parameter were extracted. Significant differences were determined based on the non-overlap of 95% Highest Density Intervals (HDI). If the 95% HDIs of the parameter posterior distributions under two conditions did not overlap, the parameter was considered to exhibit a significant difference.

(3) Model Comparison

Information criteria were used for model comparison, mainly including the Akaike Information Criterion (AIC) and Bayesian Information Criterion (BIC). AIC focuses on predictive ability under finite samples, while BIC places greater emphasis on penalizing model complexity. In addition, to further verify the consistency of model selection, the Leave-One-Out Cross-Validation Expected Log Predictive Density (ELPD_loo) was calculated, and all combinations of models were compared pairwise. ΔELPD (ELPD_loo of the superior model − ELPD_loo of the inferior model) was used as the effect size, and “ΔELPD > 2 × standard error” was adopted as the criterion for judging significant differences in predictive ability between models. If ΔELPD was significantly greater than zero, the superior model was considered to have significantly better predictive performance; if ΔELPD did not exceed twice the standard error, there was no significant difference in predictive ability between the two models.

#### 2.6.4. Random Forest

(1) Variable Definition

Feature Variables. Based on the above analysis, both the experimental design and computational modeling parameters affect the target variables; therefore, the parameters under these dimensions were all included as features. Specifically, since the DDM parameters (v, a, t_0_, Z_0_) were estimated at the participant × condition × block level, they were mapped to each individual trial based on the specific Rule Hierarchy and Block to which the trial belonged. This alignment allowed the Random Forest model to assess how the distinct “cognitive states” estimated by DDM explain the trial-by-trial variance in decision performance. Notably, the starting point proportion (z) was excluded from the final feature set to address multicollinearity. Since z is mathematically derived from the ratio of the absolute starting point bias (Z_0_) to the boundary separation (a) (i.e., z = Z_0_/a), including it alongside Z_0_ and a would introduce redundant information. To avoid this redundancy and ensure the interpretability of the feature importance rankings, we retained Z_0_ as the distinct representative of initial decision bias.

In addition, to exclude the impact of heterogeneity in the learning process (which cannot be captured by these static features) on the target variables, this study also calculated the dynamic learning process of each participant and included it in the feature system. Finally, 11 feature variables were formed, as shown in Table 1. To strictly prevent data leakage and ensure predictive validity, the Cumulative Moving Average (CMA) algorithm was applied using only historical data. For any given trial t, the dynamic learning process of accuracy (Acc_CMA_AllPast) and reaction time (RT_CMA_AllPast) were calculated based solely on the average of trials from 1 to t − 1. This ensures that the feature values for the current trial contained no information from the current or future trials. The Rule Switch Flag refers to encoding as 0 if the rule hierarchy of the current trial is consistent with that of the previous trial, and as 1 if inconsistent; it is used to identify whether a rule hierarchy switch has occurred.

Target Variables. The target variable of the classification model was trial accuracy (Accuracy), using binary encoding (0 for incorrect trials and 1 for correct trials). The target variable of the regression model was the log-transformed value of reaction time (Reaction_Time) for correct trials.

(2) Model Construction

This study used a Random Forest algorithm with pure serial implementation to construct a classification model and a regression model, respectively. The classification model performed node splitting based on Gini Impurity. During sampling, independent sample sets were generated via bootstrap, and all features were randomly selected. Regularization controlled overfitting by limiting the maximum tree depth (all set to 10) and the minimum number of leaf nodes (all set to 5).

The regression model performed splitting based on variance reduction. During sampling, independent sample sets were generated via bootstrap, and one-third of the features (based on the total number of features) were randomly selected to reduce inter-tree correlation. Other parameters were consistent with those of the classification model.

(3) Model Performance Evaluation

Two complementary strategies (Group K-Fold Cross-Validation and Out-of-Bag Evaluation) were used to evaluate the generalization ability of the models. Group K-Fold Cross-Validation dynamically partitioned the data to ensure independence between the training and test sets. Specifically, the data splitting was conducted at the participant level (ensuring that the training and test sets contained completely distinct individuals), with K set to 5. In each round of validation, data from 80% of the participants formed the training set, and the remaining 20% formed the test set. For the classification task, the accuracy index was defined as “the proportion of correctly predicted trials to the total number of trials”; for the regression task, the Root Mean Square Error (RMSE) of the predicted log-transformed reaction time (log(Reaction_Time)) for correct trials was used as the evaluation index.

Out-of-Bag (OOB) Evaluation: There was no need to separately partition a test set; instead, the “OOB samples” (not selected by bootstrap sampling during model training) were directly used to evaluate generalization ability. For the classification task, OOB accuracy was calculated; for the regression task, OOB-RMSE was calculated.

To verify the effectiveness of feature selection, a “full-feature model” (using all 11 features) and a “Top8 feature model” (selecting the top 8 features based on classification permutation importance) were trained separately. By comparing the differences in evaluation indicators between the two types of models, the impact of feature selection on model performance was verified.

(4) Model Importance Analysis

Permutation Importance was used to quantify the contribution of each feature to the model. The importance of a feature was measured by randomly permuting the values of that feature in the OOB samples and calculating the change in model performance after permutation. Each feature was permuted repeatedly 50 times, and the average performance change was taken as the final score, which was then subjected to normalization processing.

## 3. Results

### 3.1. Data Collation

A total of 40 sets of data were collected in the experiment. In accordance with the criteria, 6 sets of data were excluded (1 set due to failure to make hierarchical responses as required, and 5 sets due to accuracy rate in the learning phase not reaching 80%). Importantly, since the learning phase occurred prior to the formal experiment, these exclusions reflected a general deficit in task acquisition rather than attrition driven by specific experimental conditions (Rule Hierarchy). Therefore, the exclusions did not differ systematically by condition. The excluded data accounted for 15% of the total data. Consequently, the final valid sample size for all subsequent analyses (ANOVA, DDM, and Random Forest) was *N* = 34.

### 3.2. The Results of Analysis of Variance (ANOVA)

When analyzing the changes in participants’ accuracy rate with blocks, it was found that the main effects of Rule Hierarchy (*F*_(1.39,45.81)_ = 37.20, *p* < 0.001, $\eta_{p}^{2}$ = 0.530) and block (*F*_(4,132)_ = 2.96, *p* = 0.022, $\eta_{p}^{2}$ = 0.082) were both significant, while their interaction effect was not significant. Pairwise comparisons revealed that the accuracy rate across different rule hierarchies followed the order: Hierarchy 1 > Hierarchy 2 > Hierarchy 3, with significant differences between each pair (*ps* < 0.001); the accuracy rate of block 1 was significantly lower than that of block 2 and block 4 (*ps* < 0.05), as shown in Figure 2a.

When analyzing the changes in participants’ total correct reaction time over time, it was found that the main effects of Rule Hierarchy (*F*_(1.20,45.81)_ = 334.14, *p* < 0.001, $\eta_{p}^{2}$ = 0.910) and block (*F*_(2.33,76.97)_ = 29.24, *p* < 0.001, $\eta_{p}^{2}$ = 0.470) were both significant, and their interaction effect was also significant (*F*_(4.68,154.49)_ = 7.96, *p* < 0.001, $\eta_{p}^{2}$ = 0.194). Further analysis showed that in terms of total correct reaction time, regardless of the block, the reaction time for different rule hierarchies consistently showed the order: Hierarchy 1 < Hierarchy 2 < Hierarchy 3, with significant differences between each pair (*ps* < 0.001). When the rule hierarchy was Hierarchy 1, the reaction time trend across blocks was: block 1/2 > block 3/4 > block 5 (*ps* < 0.05); when the rule hierarchy was Hierarchy 2, the trend across blocks was: block 1 > block 2 > block 3 > block 4/5 (*ps* < 0.05); when the rule hierarchy was Hierarchy 3, the trend across blocks was: block 1 > block 2/3/4 > block 5 (*ps* < 0.01), as shown in Figure 2b.

### 3.3. The Results of Drift-Diffusion Model (DDM)

#### 3.3.1. Model Fitting Results

The model fitting results of each model are shown in Table 2. Based on the selection criteria (lowest AIC and significant ΔELPD), Model 4 (DDM with Rule Hierarchy + Block + Interaction Effect) was consistently identified as the optimal model. Its core advantages are reflected in four aspects:

First, it exhibits excellent convergence. Its R-hat value (0.99997) is closest to 1, which strictly meets the core criterion of “good convergence” for MCMC sampling in Bayesian models (R-hat < 1.01), indicating that the estimation of the posterior distribution of model parameters has the best stability and reliability.

Second, it achieves the optimal balance between fitting and complexity. Given the small sample size of this study, AIC (which focuses on optimizing predictive ability under finite samples) was prioritized as the core evaluation index. While BIC favors simpler models due to its stricter penalty on complexity, it tends to penalize interaction terms heavily, risking the underfitting of subtle but theoretically important effects. Model 4 has the smallest AIC (255.80) among all candidate models, indicating the best performance in terms of information loss.

Third, the model possesses both comprehensiveness of fitting and theoretical rationality. By incorporating the “Rule Hierarchy × Block” interaction effect, Model 4 can accurately capture the core data variation of “dynamic changes in rule effect with blocks” in multi-factor experiments. This advantage makes its BIC (358.42) significantly better than that of Model 3 (381.56) with the same degree of freedom. Although the BIC of Model 4 is slightly higher than that of Model 1 (283.48) and Model 2 (305.78) (which only include a single factor), considering its better AIC and ability to explain the interaction effect, combined with the multi-factor experimental design theory, its fitting comprehensiveness is superior to that of Model 1 and Model 2.

In addition, to strictly verify the robustness of the model selection, we employed the Leave-One-Out Cross-Validation (ELPD_loo) index to assess out-of-sample predictive accuracy. Model 4 (−359.87 ± 9.26) performed significantly better than Model 1 (−420.35 ± 9.41, ΔELPD = 60.48 ± 13.24) and Model 2 (−475.68 ± 10.22, ΔELPD = 115.81 ± 13.88). Crucially, while the difference between Model 4 and Model 3 was small (−362.15 ± 9.02, ΔELPD = 2.28 ± 12.91), Model 4 was selected because it captures the “Rule Hierarchy × Block” interaction. This interaction is theoretically essential for explaining how the learning process dynamically modulates the effect of task difficulty—a mechanism that Model 3 fails to represent.

#### 3.3.2. Decision Parameters of the Optimal Model

(1) Drift Rate (v)

The parameter estimation results of drift rate (v) showed that the main effect of Rule Hierarchy was significant, presenting an obvious gradient characteristic: as the Rule Hierarchy decreased, the drift rate significantly increased (See the Drift Rate (v) section in Table 3), indicating that individuals had a faster evidence accumulation rate under simple rules. Models 1 and 3 also showed a similar trend. The main effect of Block was significant: with the increase in the number of experimental blocks, the drift rate generally showed an increasing trend, with only a slight fluctuation in Block 5 due to the fatigue effect (See the Drift Rate (v) section in Table 3 for details), which indicated that individuals’ information processing efficiency gradually improved during the task execution. Model 3 also showed a similar trend.

**Figure 3:**
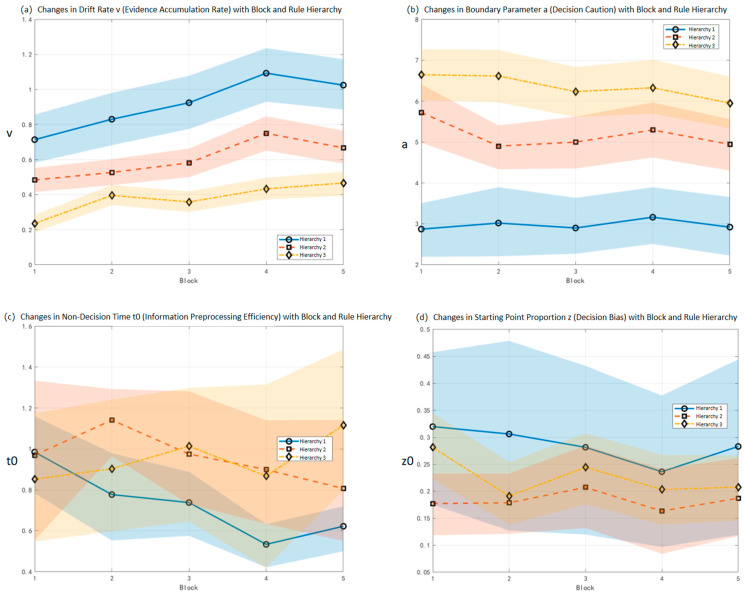
Results of Decision Parameters of the Optimal Model. (**a**) Changes in Drift Rate (v, reflecting evidence accumulation rate); (**b**) Changes in Boundary Parameter (a, reflecting decision caution); (**c**) Changes in Non-Decision Time (t_0_, reflecting information preprocessing efficiency); (**d**) Changes in Starting Point Proportion (z, reflecting decision bias). **Note:** The solid lines represent the posterior means of the parameters, and the colored shaded regions indicate the 95% Highest Density Intervals (HDI) of the posterior distributions.

The interaction effect between Rule Hierarchy and Block was significant. The interaction posterior mean was M = 0.14 (HPD = [0.05, 0.23], and the interval did not include 0), indicating that under different Block conditions, there were significant differences in the influence pattern of Rule Hierarchy on the evidence accumulation rate. The specific performance was as follows: under all Block conditions, the core law of “the lower the Rule Hierarchy, the higher the drift rate” was followed, but the difference between hierarchies showed an expanding trend with the progression of blocks. For example, in Block 1, the mean difference in drift rate between Rule Hierarchy 1 and Hierarchy 2 was 0.231 (95% HPD [0.031, 0.397]); however, in Block 4, the drift rate difference between the two was 0.343 (95% HPD [0.083, 0.453]), which was 0.112 higher than that in Block 1 (95% HPD [0.081, 0.253]). Another example: in Block 2, the mean difference in drift rate between Rule Hierarchy 2 and Hierarchy 3 was 0.134 (95% HPD [−0.02, 0.241]); while in Block 4, the drift rate difference between the two was 0.213 (95% HPD [0.153, 0.334]), which was 0.101 higher than that in Block 2 (95% HPD [0.032, 0.213]). As shown in Figure 3a.

(2) Boundary Parameter (a)

The parameter estimation results of the boundary parameter (a) showed that the main effect of Rule Hierarchy was significant, presenting an obvious gradient characteristic: as the Rule Hierarchy increased, the boundary parameter significantly increased (See the Boundary Parameter (a) section in Table 3). This indicates that when facing complex rules, individuals’ decision caution is enhanced, and they need to accumulate more evidence before making a judgment. Models 1 and 3 also showed a similar trend. The main effect of Block was significant: with the increase in the number of experimental Blocks, the boundary parameter generally showed a decreasing trend, with only a slight rebound in Block 4 due to increased task complexity (See the Boundary Parameter (a) section in Table 3 for details). This suggests that as the task execution progresses, individuals’ familiarity with the task continues to improve, their decisions gradually become more flexible, and they can complete judgments without excessive evidence accumulation. Model 3 also showed a similar trend.

The interaction effect between Rule Hierarchy and Block was not significant. The interaction posterior mean was M = −0.01 (95% HPD = [−0.04, 0.03], and the interval includes 0), indicating that under different Block conditions, there was no significant difference in the influence pattern of Rule Hierarchy on decision caution. As shown in Figure 3b.

(3) Non-Decision Time (t_0_)

The parameter estimation results of non-decision time (t_0_) showed that the main effect of Rule Hierarchy was significant, presenting a consistent trend: the non-decision time of Rule Hierarchy 1 was significantly shorter than that of Hierarchy 2 and Hierarchy 3 (See the Non-Decision Time (t_0_) section in Table 3). This indicates that under the condition of simple rules, individuals had higher efficiency in pre-decision processing stages such as information encoding, perceptual processing, and response preparation. Models 1 and 3 also showed a similar trend. The main effect of Block was also significant: with the progression of experimental Blocks, the non-decision time generally showed a gradual decreasing trend (See the Non-Decision Time (t_0_) section in Table 3), which suggests that the accumulation of task practice enhanced individuals’ familiarity with task procedures and rule requirements, thereby improving their efficiency in the preprocessing stage. Models 2 and 3 also showed a consistent trend.

The interaction effect between Rule Hierarchy and Block was not significant. The interaction posterior mean was M = 16.50 (95% HPD = [−12.40, 36.18], and the interval includes 0), indicating that under different Block conditions, there was no systematic difference in the influence pattern of Rule Hierarchy on non-decision time. The results are shown in Figure 3c.

(4) Starting Point Proportion (z)

The parameter estimation results of starting point proportion (z) showed that the main effect of Rule Hierarchy was significant, presenting an overall gradient characteristic of “the starting point proportion of Rule Hierarchy 2 is the lowest” (See the Starting Point Proportion (z) section in Table 3). This indicates that when facing Rule Hierarchy 2, individuals had the weakest bias in the initial stage of decision-making and were more likely to start the evidence accumulation process in a neutral state. Models 1 and 3 also showed a similar trend; in contrast, under the conditions of Hierarchy 1 and Hierarchy 3, individuals exhibited relatively stronger initial decision bias. The main effect of Block was also significant: with the increase in the number of experimental Blocks, the starting point proportion generally showed a decreasing trend, dropping to the lowest in Block 4 and rebounding slightly in Block 5 (possibly related to the fatigue effect) (See the Starting Point Proportion (z) section in Table 3). This result reflects that with the progression of practice, individuals’ familiarity with task rules gradually improved, their subjective bias in the initial stage of decision-making weakened gradually, and they became more dependent on objective evidence for judgment—indicating that practice has a robust weakening effect on initial decision bias. Model 3 also showed a similar trend.

Further analysis of the interaction effect revealed that the interaction posterior mean of starting point proportion z was M = 0.01 (95% HPD = [−0.02, 0.04]). Since the 95% HPD interval includes 0, it indicates that the interaction effect between Rule Hierarchy and Block on z was not significant, meaning that the influence pattern of Rule Hierarchy on initial decision bias was basically consistent under different Block conditions. The results are shown in Figure 3d.

### 3.4. The Results of Random Forest

#### 3.4.1. Accuracy Classification Model

(1) Model Performance

Results of the training set: The Full-Feature Model (with 11 core features) achieved a classification accuracy of 0.92 ± 0.01 and an F1 score of 0.92 ± 0.01 on the training set. The confusion matrix showed that the prediction recall rate for each category was ≥0.90, indicating that the model had a high degree of fitting to the training data and could fully learn the correlation pattern between features and accuracy. The Top8 Feature Model (screened based on Permutation OOB Importance) had a training set accuracy of 0.93 ± 0.01 and an F1 score increased to 0.93 ± 0.01. Due to the removal of redundant features which reduced noise interference, the model’s fitting accuracy to the training data was further optimized.

Results of the test set: The Full-Feature Model had an accuracy of 0.90 ± 0.01 and an F1 score of 0.90 ± 0.01 on the independent test set. The performance difference from the training set was small (difference in accuracy < 0.03), suggesting no obvious overfitting of the model. The Top8 Feature Model achieved a test set accuracy of 0.91 ± 0.01 and an F1 score of 0.91 ± 0.01, both 0.01 higher than those of the Full-Feature Model, which verified the optimization effect of feature selection on the model’s generalization ability.

Results of cross-validation/out-of-bag (OOB) evaluation: Stratified K-Fold Cross-Validation (K = 5): The Full-Feature Model had an average accuracy of 0.90 ± 0.01 with a standard deviation of only 0.01, indicating excellent consistency in the model’s predictions across different participant subsets. The average accuracy of the Top8 Feature Model increased to 0.92 ± 0.01, 0.02 higher than that of the Full-Feature Model, and the fluctuation range of accuracy across folds was stable (standard deviation = 0.01), further confirming the effectiveness of feature selection. There was no significant difference between the Top8 Feature Model and the Full-Feature Model (*p* > 0.05). Out-of-Bag (OOB) Evaluation: The OOB accuracy of the Full-Feature Model was 0.90, which was highly consistent with the cross-validation result (0.90). The OOB accuracy of the Top8 Feature Model was 0.92, with no significant difference from the Full-Feature Model (*p* > 0.05), and the deviation from the cross-validation accuracy (0.92) was controlled within 0.01. These results indicate that the model had stable generalization performance and no risk of overfitting.

(2) Key Classification Factors

**Figure 4:**
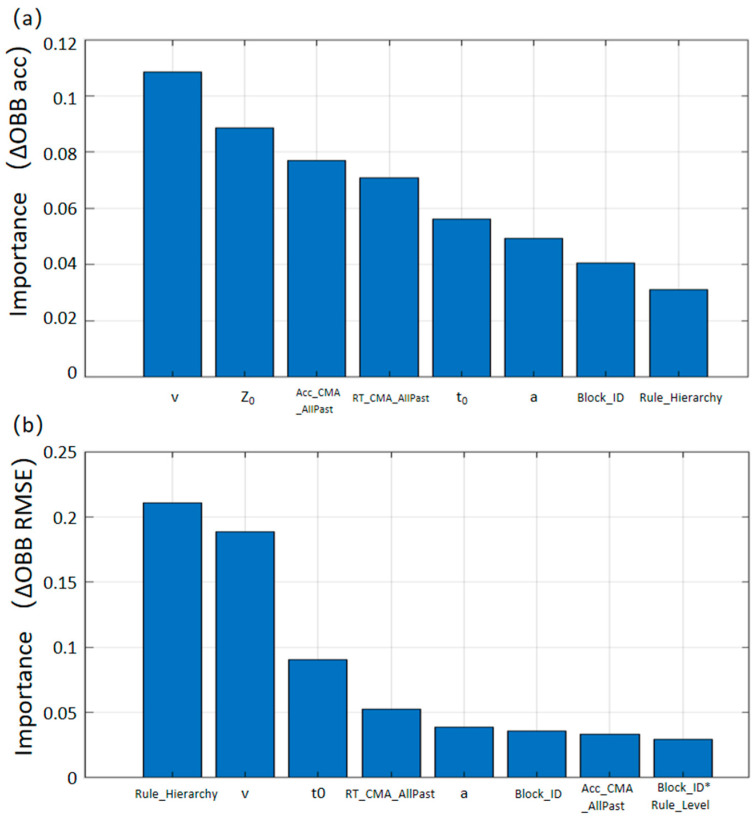
Results of the Random Forest Model. (**a**) The Top 8 features for the Classification Model (predicting trial accuracy); (**b**) The Top 8 features for the Regression Model (predicting reaction time). **Note:** The y-axis represents the Permutation Out-of-Bag (OOB) Importance. The asterisk (*) denotes the interaction term between Block Information and Rule Hierarchy.

The results of feature importance analysis were based on Permutation OOB Importance. For the classification task (predicting accuracy), the core predictors were dominated by DDM parameters and historical behavioral indicators. Among the Top 8 features, 4 were DDM parameters, with drift rate v (importance = 0.11, reflecting evidence accumulation rate) as the optimal predictor, followed by starting point bias Z_0_ (0.09), non-decision time t_0_ (0.06), and boundary parameter a (0.05). Historical behavioral indicators occupied the 3rd and 4th ranks, namely historical overall accuracy moving average (Acc_CMA_AllPast, 0.08) and historical overall reaction time moving average (RT_CMA_AllPast, 0.07), underscoring the strong predictive power of recent performance history. Additionally, the preset Block information (Block_ID, 0.04) and Rule Hierarchy (Rule_Hierarchy, 0.03) were also included in the Top 8 features, ranking 7th and 8th, respectively. The interaction term (Block_ID × Rule_Hierarchy, 0.02) ranked 9th, falling just outside the Top 8. Their importance scores were considerably lower than those of the leading DDM parameters and historical indicators. This suggests that while external task difficulty contributes to accuracy prediction, the trial-by-trial outcome relies more heavily on individuals’ endogenous decision parameters (especially information accumulation rate and initial bias) and historical performance states. The results are shown in Figure 4a.

#### 3.4.2. Reaction Time Regression Model

(1) Model Performance

Results of the training set: The Full-Feature Model (with 11 core features) achieved a predicted RMSE (Root Mean Square Error) of 0.33 ± 0.01 and a Coefficient of Determination (R^2^) of 0.80 ± 0.01 for log(Reaction_Time) on the training set. This indicates that the model could explain 79.66% of the variation in reaction time (in the log domain) in the training data, showing a good fitting effect. The Top8 Feature Model had a training set RMSE of 0.35 ± 0.01 and an R^2^ of 0.79 ± 0.01. Although there was a slight decrease compared with the Full-Feature Model (RMSE increased by 0.02 and R^2^ decreased by 0.01), it still maintained a high goodness of fit, indicating that removing low-importance features did not significantly lose the fitting information of the training data.

Results of the test set: The Full-Feature Model had a predicted RMSE of 0.35 ± 0.01 and an R^2^ of 0.73 ± 0.01 for log(Reaction_Time) for the independent test set. The difference from the training set RMSE (0.32) was controlled within 0.02, indicating that the model could still maintain good prediction accuracy on unseen data. The Top8 Feature Model had a test set RMSE of 0.37 ± 0.01 and an R^2^ of 0.73 ± 0.02. Compared with the Full-Feature Model, the RMSE and R^2^ showed no substantial change, which verified that the Top8 features had covered the core information required for reaction time prediction.

Results of cross-validation/out-of-bag (OOB) evaluation: Stratified K-Fold Cross-Validation (K = 5): The Full-Feature Model had an average RMSE of 0.35 ± 0.01 with a standard deviation of only 0.01, suggesting that the model’s prediction error on different participant subsets was controllable and highly consistent. The Top8 Feature Model had an average RMSE of 0.37 ± 0.01, which was 0.02 higher than that of the Full-Feature Model. The fluctuation range of RMSE across folds (standard deviation = 0.01) was still at a low level, and there was no significant difference from the Full-Feature Model (*p* > 0.05). Out-of-Bag (OOB) Evaluation: The OOB RMSE of the Full-Feature Model was 0.35, which was highly consistent with the cross-validation result (0.35). The OOB RMSE of the Top8 Feature Model was 0.35, with no significant difference from the Full-Feature Model (*p* > 0.05), and the deviation from the cross-validation RMSE (0.35) was negligible. This further proves that the model had stable generalization performance, and feature selection did not have a substantial impact on its core predictive ability.

(2) Key Predictive Factors

Results of feature importance analysis: Based on Permutation OOB Importance, the core predictors for the regression task (predicting log(Reaction_Time)) were as follows: Rule Hierarchy (Rule_Hierarchy, reflecting task difficulty) ranked first with an importance value of 0.21, serving as the most critical factor for reaction time prediction and directly regulating individuals’ reaction speed. The DDM parameter drift rate v (0.19, reflecting evidence accumulation rate) followed closely. These two factors together formed the “dual-core predictors,” embodying the synergistic effect of task difficulty and individual decision-making efficiency on reaction time. In addition, the interaction term between Rule Hierarchy and experimental Block (Block_ID*Rule_Hierarchy, importance = 0.03) was included in the Top8 features, suggesting that the effect of rule difficulty on reaction time changes dynamically with the progress of the experiment. In contrast, the dynamic learning indicators (RT_CMA_AllPast, Acc_CMA_AllPast) and other DDM parameters (t_0_, a, etc.) had importance values all <0.10, and only served as auxiliary predictors to supplement core information. The results are shown in Figure 4b.

## 4. Discussion

By integrating behavioral analysis, the Drift-Diffusion Model (DDM), and the Random Forest method, this study revealed the dynamic mechanism of action through which external task difficulty (Rule Hierarchy) and internal learning process (Block) influence decision performance. The results showed that: (1) Rule Hierarchy had a significant impact on both reaction time and accuracy in the early stages of the task, but this effect gradually weakened as the task progressed; (2) Internal cognitive parameters such as drift rate and boundary separation showed systematic optimization with the increase in Blocks, reflecting the dynamic improvement of cognitive processing efficiency and decision-making strategies; (3) The importance analysis of Random Forest further revealed that internal cognitive indicators had stronger explanatory power than external task difficulty in predicting decision accuracy, while external complexity mainly affected decision speed. These findings provide new evidence for understanding the interaction between task difficulty and learning process.

### 4.1. Regulation of Task Progress on the Rule Hierarchy Effect in Decision Tasks

At the behavioral level, this study found that Rule Hierarchy exhibited a stable gradient effect on decision performance: higher-level rules were associated with lower accuracy and longer reaction times. This is consistent with the conclusions of existing studies—for example, Harada et al. (2022) and Nee and Brown (2012) both pointed out that task complexity significantly increases cognitive load, leading to a decline in processing efficiency. Meanwhile, we found that this Rule Hierarchy effect gradually weakened as the task progressed, with the differences in accuracy narrowing and the differences in reaction time slowing down. This result is highly consistent with the “difficulty effect that diminishes with practice” observed by Suzuki et al. (2022) in a grammar learning task, indicating that task progress can dynamically regulate the impact of external difficulty on performance. Compared with studies such as Verbruggen et al. (2018) which only emphasized the role of rule complexity, this study further points out that external complexity is not a constant constraint, but can be weakened and regulated by learning experience.

The results of the Drift-Diffusion Model provide a mechanistic explanation for the above behavioral effects. Specifically, regarding the drift rate (v), we found a significant Rule Hierarchy × Block interaction effect: in the early stages of the task, the drift rate under high Rule Hierarchy was significantly lower than that under low-level rules, indicating that the speed of evidence accumulation was inhibited in complex tasks; however, as the number of Blocks increased, this difference gradually narrowed, reflecting that individuals could gradually improve the efficiency of evidence accumulation through experience accumulation. This means that even under complex rule conditions, individuals can optimize their processing patterns through repeated experience, gradually shifting from the initially relied-upon controlled processing to a more automatic processing process—providing direct evidence for the Active Learning Theory (Markant et al., 2016). In addition to the drift rate, other parameters also showed meaningful main effects. For non-decision time (t_0_), it showed an overall decreasing trend with the progress of the task, indicating that the efficiency of the perceptual and response preparation stages continued to improve; for starting point proportion (z), although it was relatively stable under different Rule Hierarchies, it was slightly adjusted as Blocks progressed, showing individuals’ adaptability in decision preferences and initial strategies; for boundary separation (a), an obvious main effect was observed—individuals showed greater boundary separation under high Rule Hierarchy, reflecting a more cautious decision tendency, but as experience accumulated, a gradually decreased, meaning that individuals no longer relied on excessive caution and could improve decision speed while ensuring accuracy. In summary, the interaction effect of drift rate reveals how the learning process weakens the constraints of external complexity on evidence accumulation, while the main effects of t_0_, z, and a reflect the optimization of learning in perceptual efficiency, decision preferences, and caution. This result is not only consistent with the findings of studies on motor skill learning (Moisello et al., 2009) and sequence learning (Han et al., 2024), but also indicates that the learning effect is consistent across tasks. The contribution of this study lies in further refining this “shift from controlled to automatic processing” to the level of different internal parameters, and expanding the explanatory power of the Learning Curve Theory in complex rule-based decision-making.

### 4.2. Dissociable Mechanisms of Decision Accuracy and Decision Speed

The importance analysis of Random Forest showed that drift rate, starting point bias, and dynamic learning indicators are the core factors predicting decision accuracy, while Rule Hierarchy and Block themselves have low predictive contributions to accuracy. This result suggests that accuracy depends more on an individual’s internal cognitive efficiency and learning state than on the external complexity of the task. It is crucial to distinguish here between predictive importance (as identified by Random Forest) and causal dominance (as revealed by ANOVA/DDM). While Rule Hierarchy sets the initial difficulty baseline (a causal factor), our Random Forest results indicate that accuracy is more strongly predicted by internal cognitive states (drift rate, starting point bias) and historical performance (CMA). In this context, “intrinsic learning” is not a single variable but a composite process represented by the progression of Blocks (time scale), the accumulation of trial-by-trial history (CMA), and the dynamic optimization of DDM parameters (cognitive efficiency). This suggests that maintaining high accuracy depends more on the successful adaptation of these internal mechanisms than on the external difficulty itself. This is consistent with the finding by Han et al. (2024) in sequence learning that “internal accumulation efficiency predicts accuracy,” and also addresses the limitations of traditional studies such as Harada et al. (2022): although external complexity can affect the initial performance level, the key to maintaining high-level accuracy lies in whether individuals can optimize processing efficiency through experience accumulation. It should be noted that the low importance of Block in the Random Forest model does not mean the learning effect is insignificant; instead, Block is merely a chronological indicator of experience accumulation, and its effect is mainly manifested through the regulation of internal parameters such as drift rate. Therefore, its contribution is replaced by these mechanistic indicators when predicting accuracy. Consequently, decision accuracy appears to be “state-dependent,” heavily relying on the optimization of intrinsic cognitive parameters (drift rate *v* and starting bias *Z*_0_) rather than being strictly limited by external difficulty.

Unlike accuracy, decision speed is mainly predicted by the dual factors of Rule Hierarchy and drift rate: the higher the Rule Hierarchy, the longer the reaction time; while the increase in drift rate can partially offset the delay caused by complexity. This indicates that reaction time reflects the trade-off between external complexity and internal efficiency. It should be noted that the relatively low importance of Block in Random Forest does not mean task progress has no effect on speed; instead, its effect is mainly manifested indirectly through the improvement of drift rate. In other words, as the number of Blocks increases, the speed of evidence accumulation accelerates, thereby reducing the reaction time cost caused by complexity. This finding is consistent with the proposition by Nee and Brown (2012) that “complex tasks require more evidence accumulation,” and also extends the stage model of evidence accumulation proposed by Van Maanen et al. (2021): not only does rule complexity determine the speed of evidence accumulation, but an individual’s learning process can also constantly optimize this speed. In contrast, decision speed remains “constraint-dependent,” primarily dictated by the physical complexity of the rule hierarchy, although it can be partially modulated by improved processing efficiency. This dissociation suggests a distinct “dual-mechanism” driving decision performance and cautions against viewing “decision performance” as a unitary construct regulated by a single factor.

### 4.3. Expansion of Decision-Making Theories

Synthesizing the above results, this study expands existing decision-making theories. The traditional Cognitive Load Theory emphasizes the constraints of external complexity on performance (Paas & Van Merrienboer, 2020), while the Learning Curve Theory emphasizes the improvement of performance through experience accumulation (Harris, 2022; Mazur & Hastie, 1978). This study further points out that decision performance is not solely constrained by task complexity or the number of practice trials, but is jointly determined by the dynamic interaction between the two: external task complexity determines the initial performance baseline, while the internal learning process determines the magnitude of optimization over time. This perspective not only addresses the shortcomings of the two major theories but also provides a foundation for constructing a more dynamic decision-making theory. Its value lies in two aspects: on the one hand, it helps to more comprehensively explain performance differences in complex tasks; on the other hand, it provides practical implications for the personalized optimization of educational training and human–computer interaction—for example, the burden of high-complexity tasks can be offset by strengthening the learning process.

### 4.4. Limitations and Future Directions

Although this study reveals the dynamic role of task difficulty and learning process, it still has several limitations. First, the small sample size may affect the generalizability of the results. Second, regarding the operationalization of learning, this study used Block progression as a proxy for intrinsic learning. We acknowledge that Block is a temporal variable that may conflate learning with other factors such as habituation or fatigue. However, our DDM results showed a consistent increase in drift rate (v) and a decrease in non-decision time (t_0_) across the first four blocks, which are characteristic signatures of skill acquisition rather than fatigue. Notably, the slight decline in performance observed in Block 5 suggests that fatigue began to counteract the learning effect towards the end of the task. Third, the artificially designed experimental tasks may limit the extension to real-world complex decision-making, that is, the external validity is relatively low. Furthermore, regarding model generalizability, the integration of engineered historical features (specifically Cumulative Moving Averages) was critical for capturing the dynamic trajectory of intrinsic learning. However, this approach implies a trade-off: the model’s high predictive accuracy is partly contingent on the availability of preceding behavioral data. This reliance creates a potential ‘cold start’ limitation, where the model may carry a risk of overfitting to specific historical sequences and lack immediate generalizability to the very first few trials of a new session or a new participant until sufficient history is accumulated. In addition, this study mainly relied on behavioral and modeling indicators, and has not combined neuroimaging data to explore the neural basis of how learning affects cognitive mechanisms. Future studies can expand the sample size, introduce tasks more close to real-life scenarios, and integrate methods such as EEG (electroencephalography) or fMRI (functional magnetic resonance imaging) to more comprehensively reveal the dynamic relationship among “external difficulty—internal learning—cognitive mechanisms,” and promote the application and translation of research results in education, training, and intelligent system design.

## 5. Conclusions

This study shows that although external task difficulty (Rule Hierarchy) has a significant impact on decision performance in the early stages, its effect will gradually diminish with the accumulation of practice and experience. In contrast, the improvement of internal learning process and cognitive efficiency is the key factor determining decision accuracy. Specifically, decision accuracy depends more on the optimization of intrinsic cognitive mechanisms (drift rate and bias) driven by learning, whereas reaction time remains largely constrained by the external task difficulty (Rule Hierarchy). This finding provides a new theoretical perspective for understanding the dynamic relationship between learning and decision-making, and highlights the necessity of distinguishing between these two dimensions when evaluating educational training and human–computer interaction design.

## Figures and Tables

**Table 1:** Feature Variables by Dimension.

Dimension	Feature Content
Experimental Design	Block Information (Block_ID), Rule Hierarchy (Rule_Hierarchy), Interaction between Block and Rule Hierarchy (Block_ID*Rule_Hierarchy)
Computational Modeling Parameters	Drift Rate (v), Boundary Separation (a), Non-Decision Time (t_0_), Starting Point bias (Z_0_), Model Convergence Index (Rhat)
Dynamic Learning Process	Dynamic Learning Process of Accuracy (Acc_CMA_AllPast), Dynamic Learning Process of Reaction Time (RT_CMA_AllPast), Rule Switch Flag (Rule_alt)

**Note:** The asterisk (*) denotes the interaction term between Block Information and Rule Hierarchy.

**Table 2:** Model Fitting Results of Each Model.

Model No.	Model Name	Degrees of Freedom	AIC	BIC	R-Hat	ELPD
1	DDM with Rule Hierarchy	12	262.60	283.48	0.99579	−420.35
2	DDM with Block	20	285.30	305.78	0.99666	−475.68
3	DDM with Rule Hierarchy + Block	60	258.10	381.56	0.99940	−362.15
4	DDM with Rule Hierarchy + Block + Interaction Effect	60	255.80	358.42	0.99997	−359.87

**Table 3:** Results of each parameter of drift-diffusion model.

Parameter	Effect Type	Parameter Estimate	95% Confidence Interval	Comparison with Other Models
Drift Rate (v)	Rule Hierarchy	Hierarchy 1: 0.92	Hierarchy 1: [0.85, 0.99]	All show the consistent trend of “the lower the Rule Hierarchy, the significantly higher the drift rate” with Models 1 and 3, indicating that the regulatory effect of Rule Hierarchy on information accumulation rate is robust.
		Hierarchy 2: 0.60	Hierarchy 2: [0.52, 0.68]	
		Hierarchy 3: 0.38	Hierarchy 3: [0.30, 0.46]	
	Block	Block 1: 0.48	Block 1: [0.40, 0.56]	Both show the consistent law of “the drift rate generally increases with the increase in Block” with Model 1, with only a slight fluctuation in Block 5 due to fatigue, indicating that the promoting effect of practice on information processing efficiency is robust.
		Block 2: 0.58	Block 2: [0.50, 0.66]	
		Block 3: 0.62	Block 3: [0.54, 0.70]	
		Block 4: 0.76	Block 4: [0.68, 0.84]	
		Block 5: 0.72	Block 5: [0.64, 0.80]	
	Interaction Effect	M = 0.14	M: [0.05, 0.23]	Only capturable in Model 4.
Boundary Parameter (a)	Rule Hierarchy	Hierarchy 1: 2.98	Hierarchy 1: [2.81, 3.15]	All show the consistent trend of “the higher the Rule Hierarchy, the significantly larger the Boundary Parameter” with Models 1 and 3, indicating that the regulatory effect of Rule Hierarchy on decision caution is robust.
		Hierarchy 2: 5.17	Hierarchy 2: [5.02, 5.32]	
		Hierarchy 3: 6.36	Hierarchy 3: [6.20, 6.52]	
	Block	Block 1: 5.08	Block 1: [4.92, 5.24]	Both show the consistent law of “the Boundary Parameter generally shows a decreasing trend with the increase in Block” with Model 3, with only a slight rebound in Block 4, indicating that the promoting effect of practice on decision flexibility is robust.
		Block 2: 4.85	Block 2: [4.69, 5.01]	
		Block 3: 4.71	Block 3: [4.55, 4.87]	
		Block 4: 4.93	Block 4: [4.77, 5.09]	
		Block 5: 4.61	Block 5: [4.45, 4.77]	
	Interaction Effect	M = −0.01	M: [−0.04, 0.03]	Only capturable in Model 4.
Non-Decision Time (t_0_)	Rule Hierarchy	Hierarchy 1: 731.20	Hierarchy 1: [710.50, 751.90]	All show the consistent trend of “the non-decision time of Rule Hierarchy 1 is significantly shorter than that of Hierarchy 2 and 3” with Models 1 and 3, indicating that the regulatory effect of Rule Hierarchy on information preprocessing efficiency is robust.
		Hierarchy 2: 958.40	Hierarchy 2: [936.80, 979.90]	
		Hierarchy 3: 950.60	Hierarchy 3: [928.30, 972.90]	
	Block	Block 1: 935.50	Block 1: [912.30, 958.70]	Both show the consistent law of “the non-decision time generally shows a decreasing trend with the increase in Block” with Models 2 and 3, indicating that the promoting effect of practice on the efficiency of the information preprocessing stage is robust.
		Block 2: 940.70	Block 2: [917.50, 963.90]	
		Block 3: 908.90	Block 3: [885.70, 932.10]	
		Block 4: 766.80	Block 4: [743.60, 789.00]	
		Block 5: 848.30	Block 5: [825.10, 871.50]	
	Interaction Effect	M = 16.50	M: [−12.40, 36.18]	Only capturable in Model 4.
Starting Point Proportion (z)	Rule Hierarchy	Hierarchy 1: 0.29	Hierarchy 1: [0.26, 0.32]	All show the trend that the starting point proportion z of Hierarchy 2 is the smallest with Models 1 and 3.
		Hierarchy 2: 0.18	Hierarchy 2: [0.15, 0.21]	
		Hierarchy 3: 0.23	Hierarchy 3: [0.20, 0.26]	
	Block	Block 1: 0.26	Block 1: [0.23, 0.29]	Both show the consistent law of “the decision starting point proportion generally decreases with the increase in Block” with Model 3, indicating that the weakening effect of practice on initial decision bias is robust.
		Block 2: 0.23	Block 2: [0.20, 0.26]	
		Block 3: 0.24	Block 3: [0.21, 0.27]	
		Block 4: 0.20	Block 4: [0.17, 0.23]	
		Block 5: 0.23	Block 5: [0.20, 0.26]	
	Interaction Effect	M = 0.01	M: [−0.02, 0.04]	Only capturable in Model 4.

## Data Availability

Data cannot be made publicly available because this would violate the confidentiality agreement in the informed consent. Data may be obtained from the corresponding author upon reasonable request, but cannot be uploaded publicly.
